# A new Bayesian method for the estimation of emergency nurses’ thresholds and agreement in the context of telephone triage

**DOI:** 10.3389/fpsyg.2025.1477844

**Published:** 2025-02-04

**Authors:** Michele Vicovaro, Giuseppe Mignemi, Massimo Nucci, Luigi Bolognani, Sara Iannattone, Giovanni Bruno, Andrea Spoto

**Affiliations:** ^1^Department of General Psychology, University of Padova, Padova, Italy; ^2^Emergency Medical Services (SUEM 118), AULSS 3 Serenissma, Venice, Italy; ^3^SCUP, University of Padova, Padova, Italy

**Keywords:** telephone triage, emergency, nursing, Bayesian estimation, inter-rater agreement

## Abstract

**Introduction:**

Triage is the process aimed at ensuring that patients receive a level and quality of care matching the urgency of their conditions. The present study focuses on telephone triage. We discuss the application of a new decision-making model to the task of telephone triage.

**Methods:**

The model allows to estimate the nurse’s Belonging Threshold (BT), which quantifies the minimum level of severity of an emergency scenario that leads the nurse to activate a rescue vehicle with emergency devices. The BT can be used as an index of the possible tendency of the nurse to systematically over-or under-triage. The model also provides accurate estimations of the level of agreement between different nurses, and between the nurses and reference experts, net of the noise due to the possible differences between the nurses’ BTs.

**Results and discussion:**

The model and the related experimental procedure were applied to a sample of 21 emergency nurses at the SUEM 118 Operations Center in Venice. We discuss how the model can be useful to identify nurses who would benefit from a training to improve the consistency of their application of the protocol, as well as to identify specific emergency scenarios for which the assignment of priority codes was most problematic.

## 1 Introduction

In the medical field, triage is the process aimed at ensuring that patients receive a level and quality of care matching the urgency of their conditions (Robertson-Steel, 2006). In Italy, there are three different but interrelated triage modalities: the telephone triage, the on-site procedure, and the triage carried out when the patient arrives at the Emergency Room (Ministero della Salute, 2013).

The present study focuses on telephone triage, which is the first stage of the emergency medical dispatch system (henceforth dispatch system). It is a crucial activity of the incident room aimed at evaluating the urgency of the emergency situation and selecting the appropriate rescue vehicle. When properly conducted, it prevents the under-or over-estimation of the urgency with which the patient’s health condition should be addressed. It plays a fundamental role for the prevention of medical negligence as well as undue access to the healthcare system (Ministero della Salute, 2006).

Each country has its own dispatch system, and many of these are “systematized interrogations,” based on a standardized question-and-answer logic. For instance, the Advanced Medical Priority Dispatch System (AMPDS), Criteria-Based Dispatch or NHS Pathways are different common protocols widely used is the United States, United Kingdom, and several other countries (e.g., Andersen et al., 2013; NHS England, 2014). According to these protocols, the caller’s request is assigned a specific urgency level. Each protocol might admit different questions to guarantee the emergency response accuracy and to provide the operators with the greatest possible amount of useful information. In the light of the potential life-saving role of telephone triage, it cannot be surprising that much effort that has been devoted to the improvement of the accuracy of the related protocols. To this end, various countries issue updated guidelines and promote continuous training of operators.

The Italian national dispatch system is based on a protocol-driven approach. It consists of four essential steps: (1) conducting the telephone interview, (2) giving instructions to callers, (3) properly dispatching the emergency medical service resources according to the incident priority, and (4) giving information support to rescuers until they arrive at the event location (Palma et al., 2014). To establish the priority level of the emergency situation, the nurses working at the incident room have to gather information about the location and dynamics of the event, number of individuals involved, and health condition of the injured (Ministero della Salute, 2006). Based on the preliminary evaluation of the patient’s health condition, the emergency nurses attribute a priority code. The codes are stipulated in the Ministerial Decree of 1992 (see Table 1): the red code indicates that the patient is in critical, life-threatening conditions and needs immediate access to healthcare; the yellow code specifies a potentially life-threatening condition which could worsen without an immediate intervention; green and white codes represent non-life-threatening conditions. Specifically, green code is assigned to patients who do not need an urgent examination and whose conditions are hardly critical, whereas white code is assigned in situations that could potentially be solved by general practitioners and for which a rescue vehicle would not be necessary (Guidetti et al., 1999; Leopardi and Sommacampagna, 2013; Ministero della Salute, 2013). The rescue vehicle moves urgently (i.e., with emergency devices) only when the emergency nurse assigns a yellow or red code.

**Table 1:** Description of the priority codes according to the Italian dispatch system (Dgr n. 1,298, 16 August 2017).

Red code	Ongoing impairment of vital functions or rapidly evolving pathology; involves the immediate sending of the highest level of assistance available.
Yellow code	Potentially developmental pathology in the short term or high conditions risk/inconvenience for the user; involves the immediate sending of an adequate level of care for user needs.
Green code	Absence of risk of evolution in the short term and of the need for immediate help; however, it requires access to the emergency room quickly.
White code	It does not require quick access to the emergency room; transportation can be scheduled in the following hours, based on the availability of vehicles and/or services at the reference hospital.

The dispatch system is crucial for patient’s safety (Dippenaar, 2020; Hinson et al., 2019). To guarantee its reliability, healthcare companies and institutions are required to prepare clinical and organizational protocols grounded on evidence-based medicine and evidence-based nursing (e.g., Choi et al., 2021; Jagtenberg et al., 2017; Tamburlini et al., 1999; Wallace et al., 2020; Widgren and Jourak, 2011). For instance, the Italian Ministry of Health published the Recommendation n.15, which is aimed at encouraging the adoption of measures to prevent adverse events and minimize the negative effects of an incorrect attribution of the triage code (Ministero della Salute, 2013).

Despite the existence of specific protocols, making the appropriate decision in emergency situations is far from being a trivial task. Emergency nurses have to make crucial decisions in a short time based on information which is often incomplete, confused, and difficult to interpret. Moreover, nurses are aware that underestimating the patient’s conditions may have negative consequences for patient’s safety. This can explain their tendency to over-triage (i.e., to overestimate the severity of clinical scenarios, see also Buschhorn et al., 2013; Dippenaar, 2020; Göransson et al., 2005). However, systematic over-triage can be problematic because it may lead to the saturation of the available resources, making them unavailable in case of sudden, unexpected need (Dippenaar, 2020; Göransson et al., 2005).

### 1.1 Interrater agreement in the dispatch system

The uniform application of the protocol is fundamental for the efficiency of the dispatch system. According to the protocol, emergency situations should be managed in a uniform manner independently of the specific operator who answers the call. For the sake of the consistency of the dispatch system, nurses should apply the protocol, thus converging on the same priority code in the vast majority of the emergency situations. An efficient telephone triage system requires a high level of agreement among the emergency nurses (Mistry et al., 2018). Decisions driven by intuitions and personal opinions, rather than on the uniform application of the protocol, are likely to lead to divergent decisions and to low levels of interrater agreement, with obvious negative consequences for the reliability of the dispatch system.

The interrater agreement can be considered as an index of the uniformity of the protocol application, and various indices are available to quantify it. In the present study we focus on Cohen’s *κ* (Cohen, 1960), which is a widely known index that quantifies the agreement between the judgments of a *pair* of raters on a set of scenarios. The value of κ falls in the range between −1 and 1. For instance, for a given pair of nurses and a given sample of emergency calls, −1 would indicate that the two nurses systematically disagree on the priority codes of each emergency call, 1 would indicate that they always agree on the priority codes of each emergency call, and 0 would indicate chance agreement (i.e., as if the two nurses assigned the priority codes at random).

Despite their commitment to the application of the protocol, nurses’ decisions can be affected by time constraints and environmental issues (e.g., overcrowding, staffing, inpatient bed availability, interruptions) as well as by personal characteristics such as education, amount of experience, and personality traits (Dateo, 2013; Garbez et al., 2011; Göransson et al., 2006; Wolf et al., 2018). For instance, nurses’ decision criteria have been shown to vary with their familiarity with the triage system (i.e., years of experience; Cone and Murray, 2002; Vatnøy et al., 2013). Experienced nurses tend to be more confident in their abilities, and to base their decisions more on previously faced cases than on a strict application of the protocol (Dallaire et al., 2012). Contextual and individual factors may reduce the level of agreement between the emergency nurses.

At an empirical level, substantial research examined interrater agreement in the triage system, with contrasting results. The measured level of interrater agreement ranges from fair-to-good (Dallaire et al., 2012), to moderate-to-substantial (Martin et al., 2014), to high (Parenti et al., 2010). As suggested by Göransson et al. (2005), these discrepancies can depend on differences in triage scales (i.e., the rules for providing the severity codes) as well as on differences in the indexes used to quantify interrater agreement (e.g., weighted or unweighted Cohen’s *κ*).

### 1.2 Belonging measure and belonging threshold in telephone triage

Although the dispatch system is one of the building blocks of healthcare systems, methods for the evaluation of its efficiency and reliability have received relatively little attention. In the present work, we discuss the application of a decision-making model developed by Nucci et al. (2021) to the task of the selection of the appropriate priority code in telephone triage. The model allows to tackle an under-appreciated issue of the telephone triage task, which is the possible difference between the nurses’ individual thresholds in the assignment of the priority codes. Estimating the nurses’ individual thresholds is important for at least three interrelated reasons. First, to evaluate a nurse’s attitude towards the triage task, such as his/her tendency to under-or over-triage; second, to increase the accuracy of inter-rater agreement estimation, by taking possible differences between the nurses’ individual thresholds into account; third, as a valuable quantitative tool for improving the continuous training of the operators, through the detection of their general attitude and of possible outliers.

Nucci et al. (2021) model applies to any decision-making context in which a rater has to decide whether an element *i* belongs to a category *c* or not (i.e., a binary classification task). The model assumes that a rater’s overt binary decision about the belonging of *i* to *c* (i.e., “*i* belongs to *c*” vs. “*i* does not belong to *c*”) depends on two covert (i.e., unobservable) variables. The first one is the extent to which *i* belongs to *c* according to a rater *r* (i.e., BM*_icr_*: Belonging Measure of element *i* to category *c* according to a rater *r*). The BM*_icr_* can be quantified along a continuum from 0 (i.e., “*i* definitely does not belong to *c*”) to 1 (i.e., “*i* definitely belongs to *c*”). The second covert variable is the rater’s Belonging Threshold (BT*_r_*), which is defined on the same scale as the BM*_icr_*. It is a numerical value above which a rater *r* classifies *i* as belonging to *c*, and below which the same rater classifies *i* as not belonging to *c*. For instance, if BT*_r_* = 0.4, then, for any element *i* and category *c*, the rater will classify *i* as belonging to *c* whenever BM*_icr_* > 0.4 and as not belonging to *c* whenever BM*_icr_* ≤ 0.4.

The BM*_icr_* and the BT*_r_* operationalize different aspects of the decision-making process. The BM*_icr_* reflects the opinion of a rater *r* about a specific element-category pair. It depends on the raters’ knowledge and interpretation of both the element and the category. The BT*_r_* is, instead, assumed to remain constant throughout the classification task (i.e., for a given rater, it is assumed to be constant for all element-category pairs), and would reflect a relatively stable attitude of the rater (i.e., the rater’s individual threshold). In a classification task, a rater with a low BT*_r_* (e.g., 0.2) tends to classify the elements as belonging to a category even when the evidence is relatively weak (e.g., 0.3) whereas a rater with a high BT*_r_* (e.g., 0.8) tends to classify the elements as belonging to a category only when strong evidence is available (e.g., evidence of 0.7 would not be sufficient for that rater). This method has also been applied to assess inter-rater agreement in the contexts of sport refereeing (Bruno et al., 2023) and content validity analysis (Spoto et al., 2023).

In the Italian context, telephone triage can be conceived as a classification task in which the emergency nurse classifies the urgency with which patient’s health condition should be addressed into one of four possible categories (i.e., white, green, yellow, or red; see also Table 1). However, in a practical perspective, the differences between the white and green code on the one hand, and the yellow and red code on the other hand, have relatively little importance. Indeed, the white and green codes require an ordinary rescue vehicle without emergency devices, whereas the yellow and red codes require an advanced rescue vehicle with emergency devices, as well as faster intervention time. Therefore, the decision with the greatest practical effects is dichotomous in nature: “green code or less” (i.e., white or green code) or “at least yellow code” (i.e., yellow or red code).

In line with the theoretical framework of Nucci et al. (2021) model, emergency calls can be conceived as elements that have to be classified by the emergency nurses as belonging to the category “at least a yellow code” (i.e., yellow or red code) or as not belonging no it (i.e., “green code or less”). The nurse’s implicit judgment about a specific emergency situation *i* (i.e., the BM*_icr_*) can be represented as a real number in the interval between 0 (“definitely not yellow code”) and 1 (“definitely at least yellow code”). In addition, each emergency nurse *r* would have her/his own BT*_r_*. Therefore, according to the model, *r* decides “at least yellow code” if the BM*_icr_* exceeds the BT*_r_* and “green code or less” otherwise. The BT*_r_* can be conceived as an index that quantifies the emergency nurse’s attitude towards the telephone triage task. Specifically, a relatively low BT*_r_* indicates a tendency to over-triage (i.e., a relatively low level of urgency is necessary for the rater to classify an emergency situation as “at least yellow code”), whereas a relatively large BT*_r_* indicates a tendency to under-triage (i.e., a relatively high level of urgency is necessary for the rater to classify an emergency situation as “at least yellow code”).

### 1.3 A new conceptual framework for interrater agreement in telephone triage

Besides providing a conceptual framework to the emergency nurse’s attitude towards triage, Nucci et al. (2021) model also provides a novel conceptual framework for the analysis of interrater agreement. According to the model, there can be two possible types of disagreement and two possible types of agreement.

A *true disagreement* occurs when the observed disagreement between two nurses *r* and *r’* is the result of a true implicit disagreement about the extent to which an emergency situation *i* belongs to category *c* “at least yellow code.” That is, the BM differs across the two raters, so that, for instance, according to *r* there is little evidence that the emergency situation *i* requires at least a yellow code (e.g., BM*_icr_* = 0.3), whereas according to *r’* the evidence is higher (e.g., BM*_icr’_* = 0.8). Instead, *spurious disagreement* occurs when two nurses make different explicit decisions despite the fact that they implicitly agree on the extent to which an emergency situation requires “at least a yellow code” (i.e., BM*_icr_* = BM*_icr’_*). Spurious disagreements are a consequence of differences between the nurses’ BTs. For instance, suppose that BM*_icr_* = BM*_icr’_* = 0.6. If BT*_r_* = 0.3 and BT*_r’_* = 0.7, then *r* decides “at least yellow code,” whereas *r’* decides “green code or less.” In other words, an explicit disagreement occurs despite the fact the two nurses implicitly agree about the severity of the emergency situation.

As regards the two types of agreement, *true agreement* refers to an observed agreement that results from an implicit agreement. For instance, two emergency nurses may agree that the evidence of severity of an emergency situation is 0.6. Instead, *spurious agreement* occurs when an explicit agreement is observed despite the fact that the two nurses implicitly disagree. Like spurious disagreements, spurious agreements are a consequence of differences between the nurses’ BTs. For instance, suppose that, for a given emergency call, BM*_icr_* = 0.3 and BM*_icr’_* = 0.7. If BT*_r_* = 0.2 and BT*_r’_* = 0.6, then both nurses decide “at least yellow code,” despite the fact that they clearly disagree about the extent to which the emergency call belongs to that category.

Besides indicating critical differences in nurses’ attitudes towards triage, large differences between the nurses’ BTs are cause of concern for the accuracy of interrater agreement estimation. A large number of spurious disagreements would cause the underestimation of the actual level of agreement between the nurses, whereas a large number of spurious agreements would cause its overestimation. In other words, spurious agreements and disagreements may have a detrimental effect on the reliability of interrater agreement estimation.

In this regard, Nucci et al. (2021) showed that 0.5 is the optimal value of the BT that minimizes spurious agreements and disagreements (for a detailed discussion see the seminal article). Such optimal value of the BT is defined as the Standard Belonging Threshold (*_std_* BT). If all raters had BT = *_std_* BT, then the impact of spurious agreements and disagreements on the estimated indices of interrater agreement would be minimal. In other words, if BTs = *_std_* BT then the observed level of interrater agreement would be representative of the actual level of implicit agreement among the raters. In the context of triage, BTs = *_std_* BT is highly desirable also because it would indicate the lack of a systematic tendency to under-or over-triage.

### 1.4 Agreement with the reference experts

A high level of interrater agreement between the emergency nurses is necessary, but not sufficient to guarantee the efficiency of the telephone triage system. Not only nurses should agree with each other about the priority codes, they should also agree on the *correct* codes. That is, their decisions should be appropriate for the urgency of the patient’s condition. The telephone triage system would fail if the emergency nurses tended to agree on priority codes that do not match the optimal, correct priority codes.

In the theoretical framework of Nucci et al. (2021) model, the *reference experts* are conceived as raters whose judgments represent the gold standard in a given context. Although the definition of reference expert is somewhat arbitrary and context-dependent, in telephone triage it may correspond to the heads of emergency nurses or to the professionals who are responsible for the correct application of the protocol (if different from the former). The reference experts’ judgments are assumed to be fully consistent with the protocol. Therefore, the agreement between the emergency nurses and the reference experts provides an indirect measure of the agreement between the emergency nurses and the protocol.

By definition, the reference experts’ BTs are equal to 0.5 (i.e., they correspond to the *_std_* BT; see Nucci et al., 2021). As discussed above, the *_std_* BT minimizes spurious agreements and disagreements between the nurses. It also minimizes spurious agreements and disagreements between the nurses and the reference experts. Moreover, BT*_r_* = *_std_* BT would indicate that nurse *r* has the same threshold as the reference experts, and therefore that she/he does not show any systematic tendency to over-or under-triage (although she/he might disagree with the reference experts in some scenarios). By contrast, BTs clearly smaller or clearly larger than 0.5 indicate a systematic tendency towards over-and under-triage, respectively.

### 1.5 Estimating BTs, BMs, and interrater agreement in a sample of emergency nurses: an empirical study

We illustrate the main features and advantages of the application of Nucci et al. (2021) model through an empirical example. We used the model to estimate BTs, BMs, and interrater agreement in a sample of emergency nurses working at the SUEM 118 Operations Center in Venice, Italy (SUEM stands for Servizio Sanitario di Urgenza ed Emergenza Medica; Medical Emergency and Urgency Healthcare Service).

In the healthcare system of the Veneto region, SUEM 118 manages and coordinates all the rescue vehicles, including ambulances, water ambulances, and air ambulances. It comprises various operations centers distributed across the region. The operations center of Venice is particularly important, both because of the relatively large population of the province (about 850,000 inhabitants) and because of the large flows of tourism that characterize the historical city.

The main objectives of the study can be described as follows.

1. To estimate the BT of each nurse.
2. To assess *ex-post* the predictivity of the estimated BTs, by calculating the correlation between the nurses’ BTs and the percentage of cases in which they categorized calls as “less than yellow code” (i.e., white or green code) during the year preceding the study.
3. To obtain accurate estimates of the interrater agreement between the nurses and between the nurses and a reference experts, net of possible spurious agreements and disagreements.

The estimated BTs (point 1) provide simple numerical indices that quantify the nurses’ possible tendency to over-or under-triage. The reliability of the BT as an index of nurses’ attitude in everyday triage is tested through the ex-post analysis of the relationship between the nurses’ estimated BTs and the statistics of assignment of priority codes (point 2). Moreover, an exhaustive picture of the reliability of the dispatch system as a whole, as well as a detailed picture of its reliability for specific emergency situations, is provided by the interrater agreement analysis (point 3).

An outline of the computational procedures used to estimate the BTs, the BMs and the interrater agreement will be provided in the Materials and Methods section below.

## 2 Materials and methods

### 2.1 Participants

The study was conducted in accordance with the Declaration of Helsinki, and the research protocol was approved by the Psychology Ethical Committee of the University of Padova. All participants took part in the study on a voluntary basis and provided their written informed consent before starting the experimental session. They were informed by one of the heads of the operations center that the purpose of the study was testing a new method for the evaluation of the level of agreement between emergency nurses in the assignment of priority codes. They were also informed about their right to withdraw from the study without being penalized and allowed to ask for feedback about their performance in the experimental task.

All operators of the SUEM 118 Operations Center in Venice (Italy) were invited to participate in the research and the majority accepted. A convenient sample of 21 emergency nurses (8 females, *Mean age* = 46.43 years, *SD* = 8.59 years, range = 23–62) employed in the same operations center in Venice took part in the study. Two reference experts also took part in the study. They were experienced emergency nurses and the reference for the correct application of the protocol in the operations center. The choice to involve two different independent reference experts is due to reliability and accuracy issues. Their independent judgments showed a Spearman *ρ* correlation of 0.87 (*p* < 0.001), suggesting a strong coherence between their respective opinions (data available in supplementary materials). The two independent judgments (ranging each between 0 and 10) were averaged, so to have a single reference judgment for each scenario. When a non-integer value was obtained, it was arbitrarily rounded to the nearest larger integer to simulate the behavior of an expert responding on a Likert scale using integer values. We assume that these averaged judgments are more reliable than the judgment from a single reference expert.

### 2.2 Stimuli and apparatus

The recordings of 25 emergency calls (*Mean duration* = 95.8 s, *SD* = 35.9 s, *Range* = 50–173 s) were selected by one of the authors (LB) from the archive of the operations center. Each recording contained the full dialogues between the caller and the operator, although, for privacy reasons, they were deprived of the personal data of the callers and patients, replaced with a silent interval (e.g., surname and address). The recordings included a variety of emergency scenarios, from road accidents to possible strokes, from suicide attempts to respiratory crises, just to make a few examples. In some cases, the assignment of the priority codes was made particularly difficult by reduced collaboration from the caller (e.g., due to emotional involvement, unfamiliarity with the situation) and/or by difficult environmental conditions (e.g., loud background noise). The urgency of the medical intervention could range from definitely not urgent to definitely urgent.

The experimental task was administered through a PC station in the operations center, equipped with a monitor, a pair of headphones, a keyboard, and a mouse device. Participants were tested individually before starting a work shift or at the end of it. Before starting the experimental task, they were asked to switch off any mobile device, sit in a comfortable position, and wear headphones. The experiment was programmed and presented using PsychoPy (Peirce et al., 2019), an open-source software designed for creating and running experiments in psychology, neuroscience, and related fields.

### 2.3 Procedure

The experimental procedures used for testing the reference experts were slightly different from those used for testing the other emergency nurses. Each reference expert was presented with the whole sample of 25 recordings. The two reference experts were tested in different days and care was taken that they could not talk to each other about the task, so to maximize the independence of their judgments. Instructions readable on the screen informed the reference expert that the task was to evaluate the priority of each of the emergency scenario on a scale from 0 (“definitely not a yellow code”) to 10 (“definitely at least a yellow code”). They were also informed that ratings greater than 5 would correspond to assigning at least a yellow code. A randomly selected recording was presented on each experimental trial, and a uniform gray screen appeared while the recording was playing. Immediately after the end of the recording, a Likert scale with the integer numbers from 0 to 10 appeared on the screen, and the reference expert had to select the appropriate number with a mouse click. Then, instructions invited the reference expert to press the spacebar when he was ready to listen the next recording.

Based on the responses of the two reference experts, a subsample of 15 recordings were selected from the initial sample (*Mean duration* = 92.5 s, *SD* = 32.5 s, *Range* = 50–166 s). The screening of this subsample of recordings was driven by the necessity to reduce the length of the experimental procedure (from about 45 to about 25 min), and by the necessity to maximize the accuracy of the estimation of the BTs and the BMs. A nearly uniform distribution of the possible levels of priority of the emergency scenarios is necessary for the accurate estimation of the relevant parameters (see Nucci et al., 2021), therefore the final subsample of recordings included almost all the possible levels of priority from 0 (“definitely not a yellow code”) to 10 (“definitely at least a yellow code”; see the first two columns in Table 2). A description of each emergency call is provided in Supplementary Table S1 on OSF.^1^

**Table 2:** The first column specifies the emergency scenario (detailed descriptions are available in Supplementary Table S1 on OSF).

Scenario ID	Reference experts’ average rating	Reference experts’ dichotomized response	Proportion of nurses agreeing with the reference experts
1	3	Green code or less	0.71
2	1	Green code or less	1.00
3	10	At least yellow code	1.00
4	10	At least yellow code	1.00
5	7	At least yellow code	0.90
6	2	Green code or less	0.71
7	5	Green code or less	0.71
8	6	At least yellow code	0.71
9	9	At least yellow code	1.00
10	2	Green code or less	0.90
11	4	Green code or less	0.81
12	6	At least yellow code	0.86
13	9	At least yellow code	0.81
14	8	At least yellow code	0.67
15	5	Green code or less	0.29

The second column presents the reference experts’ average ratings for each scenario, rounded to the nearest larger integer. The third column shows the reference experts’ dichotomized response: “green code or less” if the average rating was ≤5, or “at least yellow code” otherwise. The fourth column indicates the proportion, across all nurses, of instances where the nurses’ dichotomized decision, adjusted after applying the BM estimation and the *_std_* BT = 0.50, matched the reference experts’ dichotomized response.

The 21 emergency nurses were presented with the subsample of 15 emergency scenarios. The experimental procedure was the same as that for the reference experts, with the following exceptions. Upon the end of each recording, the nurses were asked to select, through a mouse click, the priority code that they would have assigned in that emergency scenario (i.e., white, green, yellow, or red). This was made to increase the ecological validity of the experimental task, making it similar to the nurses’ everyday triage task (we recall that the reference experts used instead an 11-point Likert scale). Consistently with the theoretical framework of Nucci et al. (2021) model, the nurses’ responses were dichotomized into “green code or less” (i.e., white and green code) and “at least yellow code” (i.e., yellow or red code).

### 2.4 Statistical approach

The statistical analyses were conducted through the software R (version 3.6.3). They were divided into three main parts. (1) The BTs and the BMs were estimated using the computational procedures developed by Nucci et al. (2021). (2) The predictivity of the BTs was tested by extracting, for each nurse, the statistics of frequency of assignment of priority codes over a year. Then, the relationship between these statistics and the estimated BTs was analyzed. (3) The interrater agreement among the nurses and between each nurse and the reference experts were calculated using Cohen’s *κ* (Cohen, 1960). For the interested reader, the remainder of this subsection provides some technical details about the computational procedures used in data analysis.

*Part 1*. In the first part of the analyses, nurses’ BTs and BMs were estimated through an adapted version of the computational procedures described by Nucci et al. (2021). In particular, while the original procedure used to estimate raters’ BTs and BMs by working on pairs of them, here the model was applied every time to one single rater/nurse. Therefore, the model was run 21 times, i.e., one per nurse. All the other aspects of Nucci et al.’s procedure were left unchanged.

For each nurse, the inputs of the model were (a) the 15 dichotomized responses provided in the experimental tasks (0 for white and green codes, 1 for yellow and red codes), and (b) the averaged responses provided by the reference experts in the corresponding emergency scenarios. While the former information was different for each nurse, representing the specific answers provided by him/her to each experimental stimulus, the latter was the same for all the nurses and represented the “informative prior” in the very same sense proposed in Nucci et al. (2021). In Bayesian statistics, a prior probability distribution represents the belief towards the true value of a certain parameter.

The mean of reference experts’ averaged responses were normalized within the interval 0–1 and used to build 15 prior probability distributions (i.e., one for each scenario). These were truncated (between 0 and 1) normal distributions centered on the normalized reference experts’ average rating for that specific emergency scenario and with 0.15 of standard deviation. We used functions written in Stan, a probabilistic programming language that implements Markov Chain Monte Carlo algorithms for Bayesian inference (Carpenter et al., 2017; Gelman et al., 2015; Stan Development Team, 2018), to combine the 15 priors with the 15 dichotomized responses provided by each nurse. The Stan models, relying on Bayes’ original formula to adjust their estimates, sample from increasingly probable regions of the parametric space and eventually converge towards the optimal value. For a more detailed introduction to the algorithms behind Bayesian parameter estimation using Markov Chain Monte Carlo, see Lambert (2018).

A set of functions for the estimation of BTs and the BMs are available at the following link, together with the results of the parameters estimation procedures: https://osf.io/6dwz8/?view_only=eeac939df26b4503bec19fc64bfc87fd.

*Part 2*. In the second part of the analyses, a Spearman’s rank correlation test was conducted to test whether the BTs estimated through the computational procedures just described could predict the percentage of white and green codes assigned by each nurse in the year 2019. This percentage was calculated on the total of the emergency calls managed by that nurse. A positive relationship would confirm the predictive role of the BT as concerns the nurse’s behavior in everyday triage tasks (i.e., the higher the BT, the stronger the tendency to assign a large number of white and green codes). As a side note, it is worth noting that all the nurses participating in this study worked in the same operations center and encountered similar types of emergency calls. Thus, any systematic inter-individual differences in the assignment of priority codes in 2019 are likely attributable to variability in individual characteristics and familiarity with the protocol, rather than differences in the types of calls they typically handled.

*Part 3*. The third part of the analyses focused on interrater agreement. Among the outputs of the Bayesian estimation procedure described in the first part of the analyses, there are the estimated BMs. Specifically, there were 15 BMs for each nurse, one for each emergency scenario. They represented, on a scale between 0 and 1, the nurse’s implicit judgments about the degree of belonging of each scenario to the category “at least yellow code,” where 0 stands for “definitely not yellow code” and 1 for “definitely at least yellow code “. In order to assess the interrater agreement net of possible spurious agreements and disagreements, the *_std_* BT was applied to the estimated BMs. Therefore, BMs smaller or equal than 0.50 were transformed into a “green code or less” decision, and BMs larger than 0.50 were transformed into an “at least yellow code” decision. In other words, the interrater agreement was calculated on the dichotomized responses obtained from the estimated BMs (i.e., from the implicit judgments of severity), rather than on the nurses’ overt raw responses. This allowed us to obtain indices of interrater agreement that reflect the implicit level of agreement between the nurses, net of possible differences between their BTs.

Cohen’s *κ* (Cohen, 1960) was used to quantify the interrater agreement between each pair of nurses, between each nurse and the whole sample of nurses, and between each nurse and the reference experts. By definition, the reference experts’ BT is equal to the *_std_* BT, therefore the judgments of the latter were also dichotomized into “green code or less” (i.e., ratings from 0 to 5) and “at least yellow code” (i.e., ratings from 6 to 10). We recall that the value of κ falls in the range between −1 (perfect disagreement) and 1 (perfect agreement), with a value of 0 indicating chance agreement. According to McHugh (2012), in healthcare and clinical research, values of κ below 0.60 imply an unacceptably low level of interrater agreement.

## 3 Results

### 3.1 BTs estimation and predictivity of the estimated BTs

**Figure 1:**
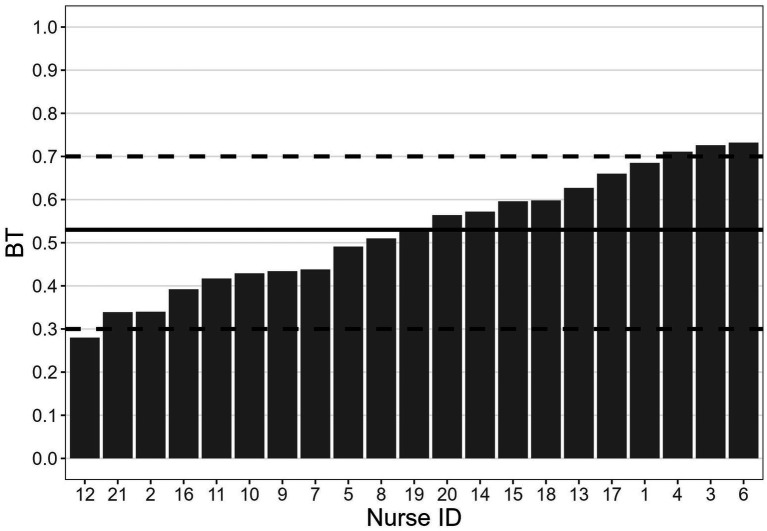
BT of each nurse. Solid horizontal line: BT sample mean; dashed lines: indicative margins for thresholds that are either too high (i.e., >0.70) or low (i.e., <0.30).

Figure 1 shows the estimated BT for each nurse. The mean BT (horizontal solid line in the graph) was 0.53 (*SE* = 0.03), very close to the ideal BT of 0.50. This result is encouraging because it indicates that, at a group level, there was no systematic tendency towards over-or under-triage. Only three operators showed a significant tendency to under-triage (BT slightly larger than 0.70, operators 4, 3 and 6), and only one operator showed a significant tendency to over-triage (BT slightly lower than 0.30, operator 12).

**Figure 2:**
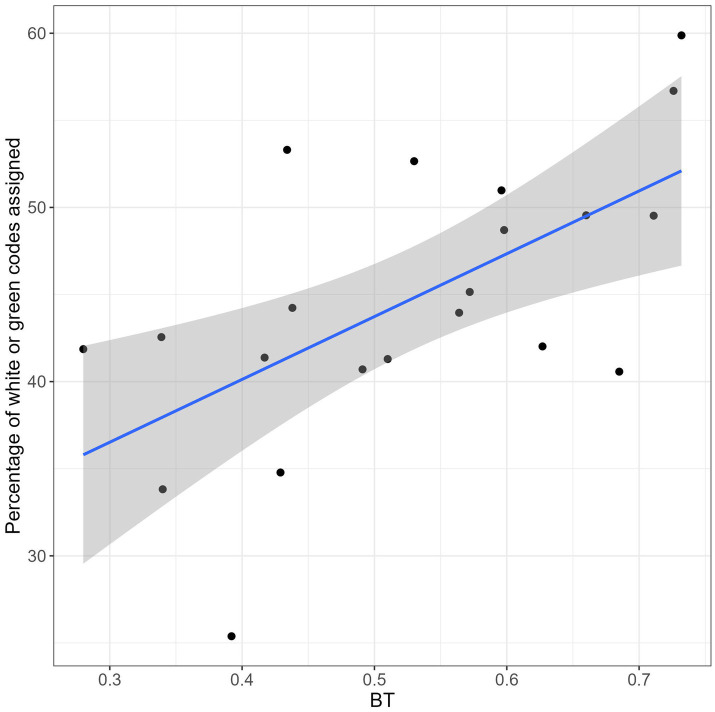
Correlation between percentage of white or green codes assigned in 2019 and BT.

The *ex-*post analysis of the 2019 data, indicative of the individual attitude in the attribution of priority codes, revealed a good uniformity among nurses. For the large majority of them, the percentage of white or green codes over the total number of codes was in the range 40–60% (see Figure 2), indicating a good balance between “green code or less” (i.e., white and green codes) and “at least yellow code” (i.e., yellow and red codes). The relationship between the estimated BT and the percentage of white or green codes assigned in 2019 was tested using a Spearman’s rank correlation test, which showed a clear positive relationship between the two variables: *ρ* = 0.58, *p* = 0.007. This result indicates that the estimated BT*_r_* predicts the nurse’s attitude in everyday telephone triage tasks: the higher the BT*_r_*, the higher the percentage of white and green codes assigned by *r* in 2019. Therefore, the BT*_r_* appears to be a reliable index of nurse’s attitude towards triage.

### 3.2 Agreement between each nurse and the reference experts

**Figure 3:**
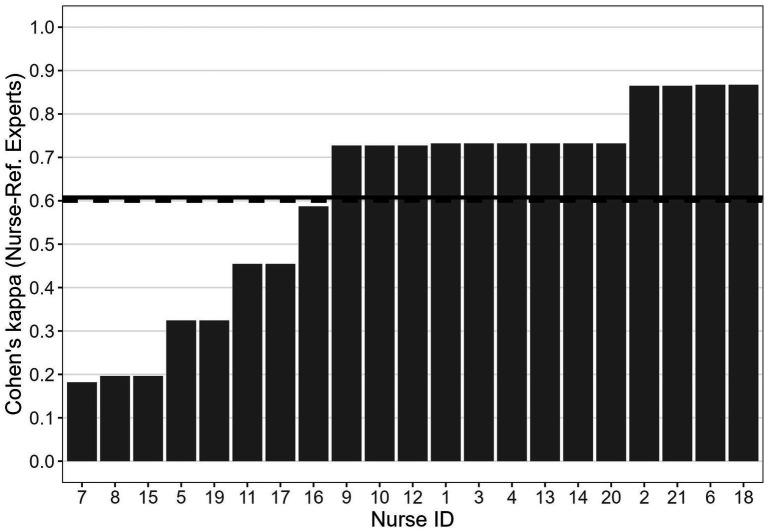
Cohen’s *κ* between each nurse and the reference experts. Solid horizontal line: mean Cohen’s κ between each nurse and the reference experts; dashed lines: indicative threshold of acceptable interrater agreement (i.e., 0.60).

Figure 3 shows the Cohen’s *κ* quantifying the agreement between the responses of each nurse and the dichotomized responses provided by the reference experts for the 15 emergency scenarios. These Cohen’s κ values do not refer to the nurses’ observed raw responses, but rather to the responses adjusted after the calculation of the individual BTs. These adjusted responses correspond to the explicit responses that the nurses would have provided had their BTs been equal to the ideal 0.50 threshold. We recall that, to compute the agreement with the reference experts, the mean of their judgments were dichotomized in “green code or less” (ratings from 0 to 5) and “at least yellow code” (ratings from 6 to 10). We also recall that, according to McHugh (2012), in healthcare and clinical research, values of κ below 0.60 imply a low level of interrater agreement. The estimated κ was larger than 0.60 for 13 out of the 21 nurses tested in our study. A moderate level of agreement with the reference experts (i.e., 0.40 < κ ≤ 0.60) emerged for three nurses, whereas for the remaining nurses the agreement with the reference expert was fair (0.20 < κ ≤ 0.40, two nurses) or slight (i.e., 0.0 ≤ κ ≤ 0.20, three nurses). Considering the whole sample of the 21 nurses, the mean Cohen’s κ was 0.61 (*SE* = 0.05).

**Figure 4:**
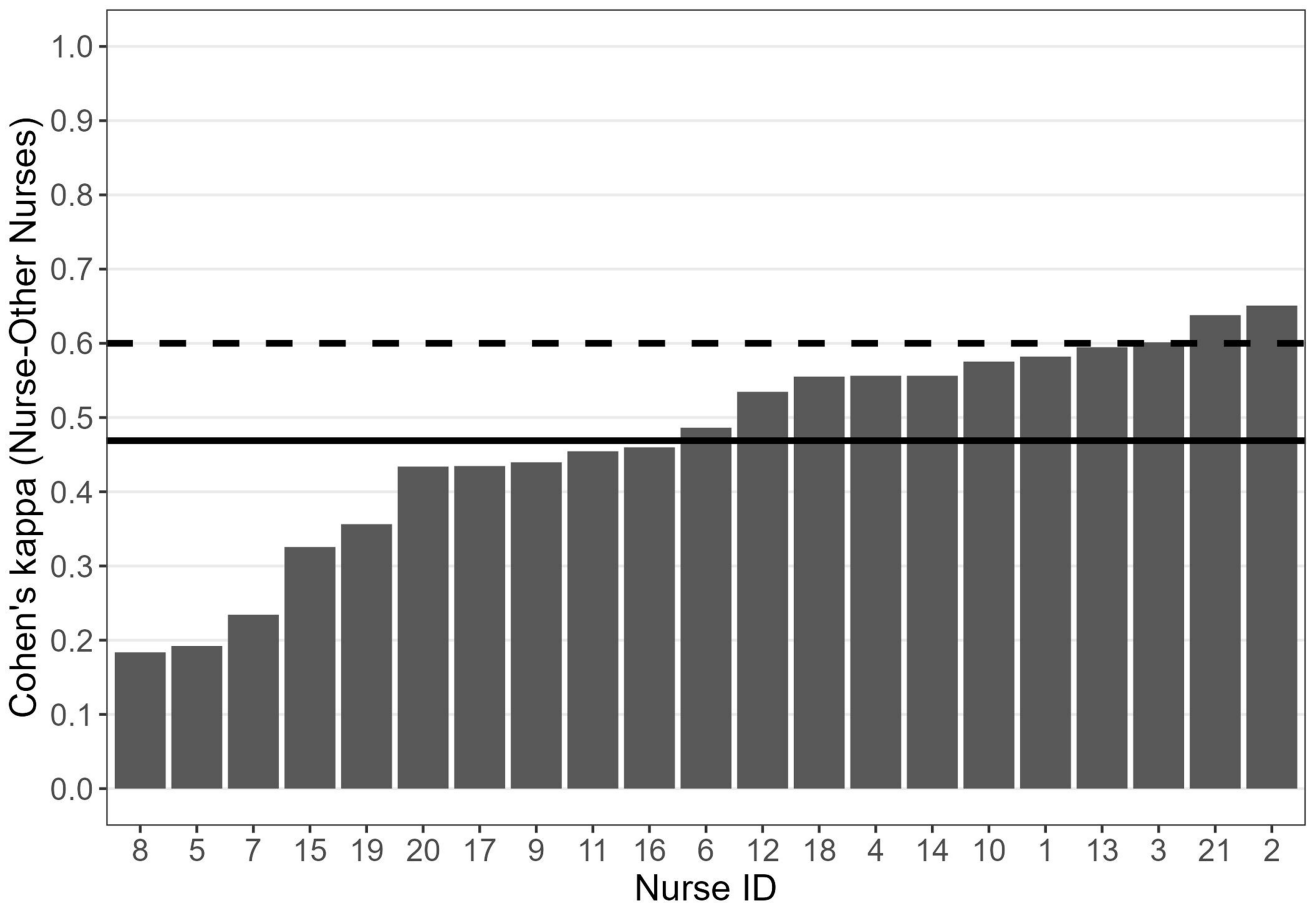
Cohen’s κ between each nurse and all the other nurses. Solid horizontal line: mean Cohen’s κ between each nurse and the other nurses; dashed lines: indicative threshold of acceptable interrater agreement (i.e., 0.60).

It is worth highlighting two interesting points about the relationships between the estimated BTs and the estimated Cohen’s *κ*. First, the BTs of the five nurses with the lowest values of κ (i.e., 5, 7, 8, 15 and 19; see Figure 3) were in the range 0.44–0.60 (i.e., close to the ideal 0.50 threshold; see Figure 1). This indicates that a BT close to 0.50, which indicates no systematic tendency to under-or over-triage, is not necessarily associated with a high level of agreement with the reference experts. Second, the BTs of three of the four nurses with the highest values of agreement with the colleagues (i.e., nurses 2, 3, and 21; see Figure 4), who also exhibited high agreement with the reference experts (Figure 3) were relatively far from the ideal 0.50 threshold (i.e., nurses 21 and 2 tended to over-triage, nurse 3 tended to under-triage; see Figure 1). These apparently paradoxical results can be explained as follows. In the present theoretical framework, the agreement with the reference experts is not calculated using the observed raw responses (which are affected by the BT), but rather using the responses corrected on the basis of the estimated BT. Notably, when the agreement with the reference experts is calculated based on the observed raw responses rather than corrected responses, it falls below the conventional 0.60 threshold for nurse 2 (from 0.865 to 0.587) and for nurse 3 (from 0.732 to 0.348). For nurse 21, it decreases but it still remains larger than 0.60 (from 0.864 to 0.727). For nurses 2 and 3, this difference between low-to-moderate agreement when observed raw responses are considered and high agreement when BT-adjusted responses are considered indicates that the implicit judgments of these two nurses, that is their BMs, align quite well with those of the reference experts, despite their BTs being quite far from the ideal 0.50 threshold.

In striving for a homogeneous triage system, the two paradoxical cases just described are concerning for different reasons. Nurses 5, 7, 8, 15, and 19, with a BT close to 0.50 but low estimated Cohen’s *κ*, would benefit from training focused on improving their evaluations of specific emergency situations. Their low inter-rater agreement, despite a BT near 0.50, indicates that their implicit urgency judgments frequently diverge from those of the reference experts. Conversely, nurses 2 and 3, with high estimated BT-adjusted Cohen’s *κ* but a BT far from 0.50, would benefit from training aimed at standardizing their thresholds (i.e., a training aimed at bringing their BTs to 0.5 or close to this value). Addressing their systematic tendencies to over-or under-triage would eliminate the need for further training on specific emergency situations, as their agreement with reference experts on implicit urgency judgments is estimated to be already high. The capacity to tailor training programs is an advantage made possible by independently measuring raters’ thresholds and inter-rater agreement. Finally, we highlight the commendable performance of nurses such as 14, 18, and 20, who exhibit both a BT close to 0.50 and high agreement with reference experts. These nurses apply the protocol optimally and require no additional training.

### 3.3 Agreement between each nurse and the other nurses

Figure 4 shows the mean Cohen’s κ quantifying the level of agreement of each nurse with all the other nurses. This value was obtained by first computing, for each nurse, the Cohen’s κ for all the 20 pairs of nurses involving that specific nurse, and then by averaging these 20 values. The mean Cohen’s κ was 0.47 (*SE* = 0.03), which indicates, overall, a relatively low level of agreement between each nurse and the colleagues. As shown by the analysis of the agreement between each nurse and the reference experts, the majority of the nurses showed a fairly high level of agreement with the reference experts (see Figure 3). It is reasonable to hypothesize that the nurses with a high level of agreement with the reference experts may also show a high level of agreement with the majority of the colleagues. This was confirmed by a Spearman’s rank correlation test, which showed a clear positive relationship between the Cohen’s κ between each nurse and the reference experts and the Cohen’s κ between each nurse and all the other nurses, *ρ* = 0.78, *p* < 0.001.

When the three nurses with the lowest level of agreement with the reference experts (i.e., nurses 7, 8, and 15) were excluded from the dataset, the overall mean Cohen’s κ between each nurse and all the other nurses raised from 0.47 (*SE* = 0.03) to 0.55 (*SE* = 0.03). The latter value is close to the value obtained for the agreement between each nurse and the reference experts, and close to a good level of agreement according to McHugh (2012) criteria.

### 3.4 Agreement between nurses and the reference experts for specific scenarios

To identify possible critical scenarios that may require a specific training, it is worth analyzing the agreement between nurses and the reference experts also at the level of the single scenario. The second column in Table 2 shows the average ratings provided by the reference experts to each of the 15 scenarios; the third column shows the reference experts’ dichotomized response, namely “green code or less” (i.e., no emergency devices) for ratings smaller than 6, or “at least yellow code” (i.e., emergency devices) for ratings equal or greater than 6. The fourth column represents the proportion of nurses whose dichotomized adjusted BM (i.e., “green code or less” if BM*_r_* ≤ 0.50, “at least yellow code” if BM*_r_* > 0.50) was consistent with the reference experts’ dichotomized response. In other words, the fourth column shows the proportion of agreement between the nurses and the reference experts for each scenario, net of possible spurious agreements and disagreements. To make an example, in scenario 1 the reference experts would assign a “green code or less” (rated severity of 3), and the same decision would be taken by 71% of the tested nurses. The proportion of agreement ranges from very low for scenario 15 to very high for scenarios 2, 3, 4 and 9.

**Figure 5:**
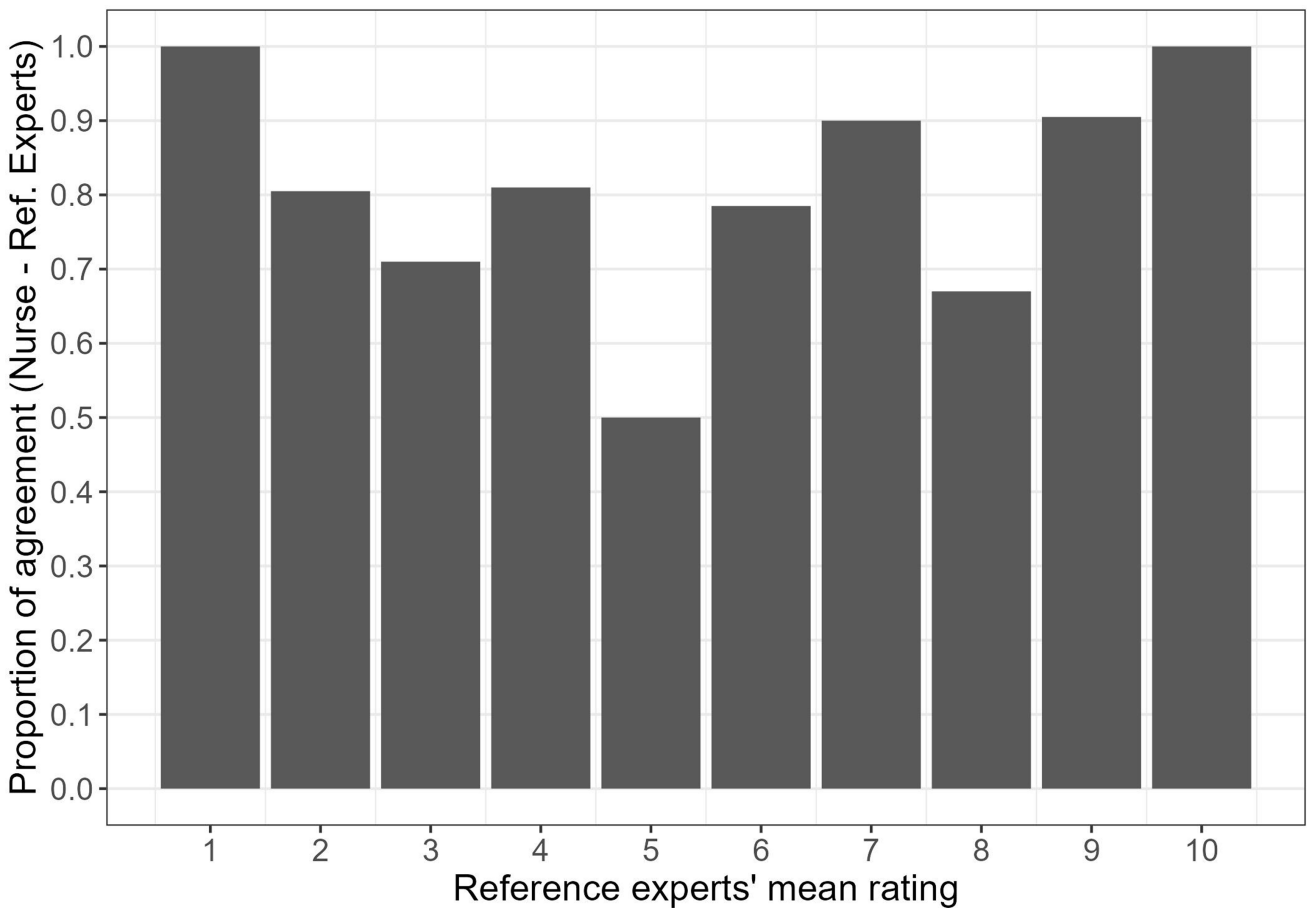
Proportion, across all nurses, of instances where the nurses’ dichotomized decision, adjusted after applying the BM estimation and the *_std_* BT = 0.50, matched the reference experts’ dichotomized response, as a function of the reference experts’ ratings of urgency of the scenario. Note that the proportions of agreement for scenarios with the same average ratings were averaged.

Figure 5 represents the proportion of agreement between the nurses and the reference experts as a function of the reference experts’ ratings. As expected, the proportion of agreement was higher for scenarios that received extreme ratings from the reference experts (i.e., scenarios with a low rated severity of 1 or 2 or scenarios with a high rated severity of 9 or 10). These extreme ratings denote that, according to the reference experts, taking a decision was relatively straightforward. Therefore, a large proportion of agreement could be expected for these scenarios. Symmetrically, a relatively low proportion of agreement could be expected for scenarios with intermediate ratings of severity, because according to the reference experts the decision was more uncertain in those cases.

Apart from the expected U-shape of the graph, some anomalous results are worthy of being mentioned. For instance, scenario 6 was assigned a severity of 2 by the reference experts, but 29% of the nurses would have assigned at least a yellow code (i.e., a significant minority of the nurses tended to over-triage in this scenario). This scenario describes an adult woman experiencing abdominal pain and vomiting episodes, who had been transported to the emergency room by ambulance the previous day for the same issues. The reference experts likely considered this latter aspect as indicative of the non-urgency of the situation. More critically, although scenario 14 was assigned a severity of 8 by the reference experts, 33% of the nurses would have assigned “green code or less” (i.e., a significant minority of the nurses tended to under-triage in this scenario). This scenario involves an elderly woman (82 years old) with a history of heart disease, complaining of an irregular heartbeat and generalized pain. The low agreement may stem from the contrast between the seemingly serious health condition and the fact that the call was made by the woman herself in a clear and loud voice. These scenarios would benefit from a specific focus by trainers of emergency nurses.

## 4 Discussion

The increasing number of emergency calls (Blodgett et al., 2021; Campagna et al., 2020) and the related risk of over-or under-triage (Bambi et al., 2021) impose a crucial issue in the emergency system. Providing accurate and efficient assessment of the required level of urgency of the medical intervention is the main task of the telephone triage (e.g., Ministero della Salute, 2006). In this regard, the correct evaluation of emergency situations is essential to ensure both the patient’s safety and a prompt response (Dippenaar, 2020; Hinson et al., 2019), and to reduce unnecessary pressures on the emergency room (Brasseur et al., 2019; Campagna et al., 2020; Rashid et al., 2021). Several protocols, methods, and tools have been developed during the last decades (Choi et al., 2021; Jagtenberg et al., 2017; Tamburlini et al., 1999; Wallace et al., 2020; Widgren and Jourak, 2011). However, making the appropriate decision in emergency situations remains a difficult task, especially because nurses’ decisions can be influenced by several environmental and individual factors (Dateo, 2013; Wolf et al., 2018).

Despite the plenty of studies and commendable progresses in this area, little attention has been devoted to possible individual systematic biases in nurses’ evaluations and to the consistency in the attribution of severity codes. This issue is addressed by the computational model proposed by Nucci et al. (2021). We used the model to estimate the nurse’s BT as his/her reference point for the binary choice between “green code or less” and “at least yellow code.” The results showed that this estimated value was strictly related to the triage evaluations over a year, providing support to the reliability of both the experimental procedure and the computational methods applied in the context of telephone triage.

The nurse’s BT provides valuable information concerning her/his attitude towards triage. A BT larger than 0.50 indicates a tendency to under-triage, whereas a BT smaller than 0.50 indicates a tendency to over-triage. As regards the sample of emergency nurses tested in the current study, the results showed that, with a few exceptions, the BTs were close to the optimal value of 0.50 (mean BT = 0.53). No systematic tendency to under-or over-triage emerged, a result which is particularly positive in the light of the fact that minimizing under-and over-triage is one of the main objectives of an efficient triage system (Buschhorn et al., 2013; Dippenaar, 2020; Göransson et al., 2005).

Another crucial advantage of the application of Nucci et al. (2021) model is the possibility to estimate the level of agreement between the emergency nurses and between the emergency nurses and the reference experts, net of possible spurious agreements and disagreements. In this regard we recall that the conformity and the accordance between different nurses are one of the most valuable objectives of the triage system (Bambi et al., 2021; Silva et al., 2017). At the group level, the agreement between the nurses and the reference experts was fairly high (mean *κ* = 0.60), whereas the agreement among the nurses was relatively low (mean κ = 0.47), although the latter value increased to 0.55 after removing the data of the three nurses with the lowest levels of agreement with the reference experts. It is worth emphasizing that a relatively high mean values of κ may conceal low values of κ for some of the raters, therefore analyzing the interrater agreement at the individual level is recommended. It is also worth analyzing the interrater agreement at the level of single scenarios (see Table 2), as this can provide heads and trainers of emergency nurses useful information about the critical scenarios that would require a specific training.

### 4.1 Limitations and further directions

The present empirical study, conducted on a sample of emergency nurses working at the SUEM 118 Operations Center in Venice, should be conceived as an illustrative example of the application of the Bayesian model and experimental procedure discussed in the introduction. Our sample was not representative of the population of emergency nurses working at telephone triage services in Italy. The results cannot be extended beyond the sample itself, therefore they do not represent a picture of the functioning of the telephone triage system in Italy. Nevertheless, this small-scale study can be useful to other researchers for the possible implementation of the methods and the computational procedures in large-scale studies aimed at providing a representative picture of the effectiveness of the telephone triage system in Italy as well as in other countries. The methods and procedures described in the present work represent a first step towards a more comprehensive assessment of the emergency nurses’ performance in the telephone triage task.

On the technical side, in the present study, we obtained independent evaluations from two reference experts and averaged them for simplicity. Given the very high correlation between their evaluations (*ρ* = 0.87), this approach had minimal impact on the results. However, in broader contexts, averaging ratings from multiple experts may introduce an artifact, particularly when there is low agreement between the reference experts, and should therefore be avoided.

It is important to underline that the accuracy of the estimated BT is conditional upon the correct application of the experimental procedure. Using a sample of emergency calls representative of all the severity continuum from 0 to 10 is necessary for an accurate estimation of the BT (see also Nucci et al., 2021). Moreover, the BTs can be correctly interpreted only if the reference experts’ judgments actually represent a gold standard. In other words, the reference experts’ ratings should reflect the *optimal* application of the protocol, otherwise the BTs cannot be correctly interpreted in terms of tendencies to under-or over-triage. A careful selection of the reference experts is thus fundamental for the correct interpretation of the BTs. This issue is a well-known and explored issue in Bayesian methods, wherein the importance of the quality of the priori information is the subject of a large amount of research.

## Data Availability

The datasets presented in this study can be found in online repositories. The names of the repository/repositories and accession number(s) can be found at: Open Science Framework. Link: https://osf.io/6dwz8/?view_only=eeac939df26b4503bec19fc64bfc87fd.

## Footnotes

^1^ https://osf.io/6dwz8/?view_only=eeac939df26b4503bec19fc64bfc87fd
